# A tutorial on oxidative stress and redox signaling with application to exercise and sedentariness

**DOI:** 10.1186/s40798-014-0003-7

**Published:** 2015-01-20

**Authors:** Robert Buresh, Kris Berg

**Affiliations:** 1 Department of Exercise Science and Sport Management, Kennesaw State University, 520 Parliament Garden Way NW, Kennesaw, GA 30144 USA; 2 School of Health, Physical Education, and Recreation, University of Nebraska at Omaha, Omaha, NE USA

## Abstract

Oxidative stress has been shown to play a role in the etiology of several chronic diseases, including cardiovascular disease, diabetes mellitus, and cancer. Free radicals and, most prominently, the superoxide radical, result from oxidative metabolism and several enzyme-catalyzed reactions, and endogenous cellular antioxidants dismutate many reactive oxygen species (ROS). Under certain conditions, ROS production can outpace dismutation (e.g., long-term sedentariness and positive energy balance) and the result is oxidative stress, with proteins, lipids, and DNA the most common targets of radicals. However, the molecules that contribute to oxidative stress also appear to participate in vital cell signaling activity that supports health and stimulates favorable adaptations to exercise training, such that inhibiting ROS formation prevents common adaptations to training. Furthermore, researchers have recently suggested that some proteins are not as readily formed when the redox state of the cell is insufficiently oxidative. Exercise training appears to optimize the redox environment by dramatically enhancing the capacity of the cell to neutralize ROS while regularly creating oxidative environments in which membrane and secretory proteins can be synthesized. The role that exercise plays in enhancing management of ROS likely explains many of the associated health benefits.

## Key points

- Reactive oxygen species (ROS) in excess are toxic and have been implicated in the development of aging and chronic disease.
- ROS have also been shown to enhance acute muscular activity, and to be required signaling molecules for favorable adaptations to exercise training.
- Long-term sedentariness is characterized by chronically elevated basal ROS production and reduced antioxidant capacity.
- The oxidative environment induced by exercise stimulates antioxidant capacity and may enhance synthesis of specific proteins.

## Introduction

Oxidative stress has been implicated in the etiology of a number of chronic diseases including cardiovascular disease, diabetes mellitus, and cancer. It has been defined as an imbalance between oxidants and antioxidants in favor of oxidants, leading to the disruption of redox signaling and control and/or molecular damage [1]. Recent studies have shed a great deal of light on factors that influence oxidative stress. Exercise training is among the factors known to provide protection against oxidative stress, and understanding how exercise training improves oxidative status may be helpful to exercise professionals. The purposes of this review are to (1) explain what oxidative stress is, and how and where it occurs; (2) summarize recent studies that suggest that some minimal level of oxidative molecules are essential for cell signaling and stimulating beneficial adaptations to training; (3) explain how oxidative agents, in excess, are damaging; and (4) explain the effects of acute exercise and chronic exercise training on oxidative stress. As the major focus of this paper is exercise, we primarily consider these concepts within muscle fibers. However, exercise appears to stimulate reductions in signs of oxidative stress systemically (e.g., in brain and liver tissue), and those influences are also addressed briefly.

## Oxidation, reduction, and radicals

Oxidation occurs when a molecule loses an electron. When one molecule loses an electron, another will acquire it, and the molecule that gains an electron is said to be reduced. Oxidation and reduction, then, occur together, in that whenever a molecule is oxidized, another is reduced. The fact that they occur together has led to the development of the term ‘redox’ being used in the context of this class of chemical reactions.

While there are countless examples of redox reactions in biochemistry, the reactions that are most closely related to oxidative stress involve molecules that are especially strong oxidizing agents, known collectively as free radicals, and include reactive oxygen species (ROS) and reactive nitrogen species (RNS) (the predominance of RNS is often referred to as nitrosative stress). These molecules are especially strong oxidizing entities because they have an unpaired electron in their outer shells. Oxygen and nitrogen are atoms that display a high level of electronegativity, owing to the size of the atoms and the number of protons in their nuclei. The result is that these atoms have a strong attraction for electrons when there are no unpaired electrons in their outer shells, and that attraction is much stronger when there is an unpaired electron.

One commonly produced radical, and one that is the basis of other oxidative agents, is the superoxide radical—molecular dioxide (O_2_)—that has undergone a one-electron reduction. Superoxide reacting with other molecules can result in other radicals or oxidizing agents being produced. For example, much of the superoxide that is produced in mitochondria is dismutated or partially neutralized by an enzyme known as manganese superoxide dismutase (MnSOD), a protein that converts superoxide to hydrogen peroxide (H_2_O_2_). H_2_O_2_ is an oxidizing agent, but it does not have an unpaired electron, so it is less reactive and much more stable than superoxide. The relative stability of H_2_O_2_ lends to its ability to serve as a cellular signal of oxidative, or redox, state, a role that is discussed later.

The hydroxyl radical is another ROS, the neutral form of the hydroxide (OH) ion. It can result from H_2_O_2_ reacting with iron, or from superoxide reacting with H_2_O_2_ [2]. Like the superoxide radical, this molecule is extremely reactive and unstable. In fact, it is more reactive than any other known radical, reacting with nearly any molecular entity typically within two molecular diameters [3].

## Biological Sources of Reactive Oxygen Species (ROS) and endogenous antioxidants

Some production of ROS appears to be an inescapable byproduct of cellular oxygen consumption, and radicals are produced in several commonly occurring biological reactions with many being quickly neutralized by various forms of antioxidants inside the cell. For example, superoxide radicals are produced in reactions of the electron transport chain (ETC.) within the mitochondria. The reduced forms of nicotinamide adenine dinucleotide (NADH) and flavin adenine dinucleotide (FADH_2_) deliver electrons to the ETC. at specifice sites, and the electrons move down the chain from carrier to carrier, releasing energy that is used to pump protons from the mitochondrial matrix to the inter-membrane space and thereby developing an electrochemical gradient that drives adenosine triphosphate (ATP) synthesis by ATP synthase. In the process of electrons being passed down the ETC., some escape, or leak, and are delivered to molecular oxygen forming superoxide. This has been shown to happen most commonly at ETC. complex I (ROS is formed within the mitochondrial matrix) and ETC. complex III (ROS formed within the mitochondrial matrix and in the inter-membrane space) [4,5]. Basal ROS production had previously been estimated to account for between 2 and 5% of oxygen consumed, but more recent evidence suggests that it is substantially less—perhaps approximately 0.15% of consumed oxygen [6,7]—and ROS production at ETC. complex I increases by approximately 187%, and at ETC. complex III by 138% during exhaustive muscular activity [7]. Note that while this is a sizeable increase in the rate of ROS production, it may be less than one might expect given that the metabolic rate of contracting muscle fibers increases approximately 100-fold or more from resting levels [8]. This suggests that mitochondria in skeletal muscle produce ROS at a much lower rate per unit of oxygen consumption during contractile activity, and one possible explanation for that is considered later.

The increase in ROS production in the mitochondria appears to be a result of increased electron flux during muscular activity, and, as noted above, much of it is reduced by superoxide dismutase (SOD) (MnSOD is found within the mitochondrial matrix, while copper- and zinc-containing SOD [Cu/ZnSOD] is primarily in the cytosol [9]) and converted to H_2_O_2_. Some of the H_2_O_2_ is further neutralized by the activity of catalase, which converts it to water and molecular oxygen. H_2_O_2_ can also be reduced to water and oxygen by other enzyme-catalyzed reactions including glutathione peroxidase and the thioredoxin systems [10-12]. However, as is discussed later, H_2_O_2_ also serves as a signaling molecule and its oxidative nature is used to trigger events in the cell.

Another muscular source of superoxide is an enzyme called nicotinamide adenine dinucleotide phosphate (NADPH) oxidase (NOX). In the context of this discussion, one important role of NADPH involves an antioxidant. Glutathione (GSH) is a molecule that is capable of dismutating ROS, itself becoming oxidized in the process. When oxidized, it will bind with another oxidized GSH molecule, resulting in its oxidized form, GSSG. In this form, it can no longer function as an antioxidant until it is reduced, and an enzyme called GSSG reductase performs that reaction using NADPH as a cofactor and producing two reduced GSH molecules that can again quench ROS [13]. As such, NADPH enhances the capacity of the cell to neutralize ROS through its involvement in reducing oxidized GSH.

The enzyme NOX is unique in that it is the only mammalian enzyme for which the sole purpose is ROS synthesis [14]. It functions by transferring an electron from NADPH to molecular oxygen, thereby forming superoxide [15]. NOX can be found in a number of locations, one of which is near sarcoplasmic reticulum (SR). NOX-produced ROS at the SR have been shown to enhance calcium release from the SR by oxidizing calcium release channels [16,17]. This NOX is stimulated by calcium release [18], and as such its activity is elevated during muscle contraction. Another site of NOX localization is at transverse tubules of skeletal muscle, where it is stimulated by action potentials [19,20], and it may be that the ROS synthesized in this area serve as a redox signal, either intra- or extracellularly. In any case, NOX enzymes produce ROS and do so at an increased rate during muscular contraction.

Phospholipase A2 (PLA2) is another enzyme that participates in ROS production, largely by activating NOX [21]. Some PLA2 isoforms are dependent on calcium and most are found within mitochondria, while other isoforms are calcium-independent and found mostly in the cell cytoplasm. It has been suggested that the calcium-dependent PLA2 is responsible for ROS formation during exercise, while the calcium-independent PLA2 is responsible for basal ROS production [22].

Xanthine oxidase (XO) also generates superoxide radicals while oxidizing hypoxanthine and forming xanthine [23]. Hypoxanthine is produced as a result of adenosine monophosphate (AMP) degradation, wherein AMP is first converted to inosine and then to hypoxanthine [24]. An increase in the availability of the substrate hypoxanthine may increase the rate of XO activity and, therefore, ROS production, and hypoxanthine levels increase with exercise, and especially with intense or anaerobic exercise. This possibility is supported by Radak et al. [25] who reported a linear relationship between intramuscular lactic acid concentration and XO activity during exhaustive treadmill exercise in rats.

Nitric oxide (NO) is a molecule that serves a number of functional and signaling roles. It is produced by one of three isoforms of NO synthase (NOS): endothelial NOS (eNOS), inducible NOS (iNOS), and neuronal NOS (nNOS). eNOS plays an important role in blood flow distribution to support muscular activity. Under optimal conditions (i.e., when all necessary substrates are available), eNOS produces NO, which then induces vasodilation via relaxation of vascular smooth muscle cells resulting in hyperemia to active muscle fibers. However, the lack of any requisite substrate for eNOS results in an ‘uncoupled’ enzyme, and in this state eNOS production of superoxide is increased [26-28]. The increase in ROS has the capacity to reduce functional NO via two related mechanisms: (1) NO can react with superoxide, which results in the formation of peroxynitrite radicals; and (2) ROS, and especially peroxynitrite, can reduce the availability of one necessary substrate of NOS, thereby further uncoupling NOS and further increasing superoxide production [26,29]. These outcomes impair the capacity for hyperemia to active skeletal muscle to support contractile activity. In addition, peroxynitrite is a particularly reactive molecule, and has been shown capable of causing oxidative injury and of triggering necrosis and apoptosis [30].

A number of additional mechanisms of ROS production are known to exist, but it appears that those described here are the most productive [31], so we confine our further discussions to these pathways. Importantly, all ROS species produced in skeletal muscle are derived from either superoxide or NO [32], and the production of, and neutralizing of, these two molecules explains most of cellular redox state at any given time.

## ROS as critical signaling molecules

As discussed above, there are a number of enzyme-catalyzed pathways through which ROS are produced. While there is considerable interest in the role that oxidative stress plays in the etiology of chronic diseases, consideration of these reaction pathways is also suggestive that a threshold level of ROS must be needed for optimal cellular function. That is especially apparent when considering NOX, as the sole known purpose for this enzyme is the production of ROS, as opposed to the other reaction pathways that have been identified wherein ROS production is a by-product.

For a molecule to function effectively in a signaling role, its levels must be able to be regulated (i.e., adjusting rates of synthesis and degradation), it must have specific targets or receptors, and its signaling effect must be reversible [33]. Superoxide and hydroxyl radicals and peroxynitrite are highly reactive and unstable, and, as such, are unsuitable in a signaling role. That is, they simply cannot travel far without oxidizing some nearby molecule. However, H_2_O_2_ is considered to be the primary intracellular redox signaling molecule [34-36]. H_2_O_2_ is small and can diffuse easily, and it is stable enough to travel and oxidize specific sites on target molecules [37]. As discussed previously, superoxide and other more reactive radicals are partially neutralized by being converted to H_2_O_2_, allowing this molecule to be reflective of the overall oxidative state in the cell.

Several studies have produced results in support of ROS having a signaling role, both with short-term exercise and as a result of exercise training. As noted above, NOX-induced ROS contribute to increasing calcium release from SR. Reid [38,39] showed that reduction of ROS below basal levels reduced the force production of skeletal muscle, while increasing ROS to a certain point increased force production, and ROS exposure above that point resulted in a reduction in force production [40]. Other studies have demonstrated that beneficial training adaptations are eliminated when trainees are treated with antioxidants. Gomez-Cabrera et al. [41] found that 1,000 mg per day of vitamin C supplementation attenuated increased PGC-1a (peroxisome proliferator-activated receptor-gamma coactivator-1 alpha; a transcriptional co-factor that is involved in initiating mitochondrial biogenesis) expression and, consequently, attenuated the increase in mitochondrial density that commonly occurs with aerobic exercise training. In addition, the training-induced increase in several antioxidant enzymes was inhibited with vitamin C supplementation [41]. Similarly, Ristow et al. [42] found that daily supplementation with a combination of 1,000 mg of vitamin C and 400 IU of vitamin E eliminated the training-induced increases in insulin sensitivity and PGC-1a expression, as well as levels of endogenous antioxidants SOD and glutathione peroxidase. It is noteworthy that another recent study of the effects of antioxidant supplementation on training adaptations in humans found that daily supplementation with 500 mg of vitamin C and 400 IU of vitamin E had no effect on physiological responses to 12 weeks of aerobic exercise training [43]. These contradictory findings may be attributable to the dosage of antioxidant supplement utilized, but further research in this area is merited [43].

Although how ROS signaling induces adaptations to training is not fully understood, a number of possible mechanisms have been identified. For example, certain proteins, especially those composed of sulfur-containing amino acids such as cysteine, are readily and reversibly oxidized by ROS [44,45]. In addition, the oxidation of amino acid residues containing aromatic structures (e.g., histidine, tyrosine, tryptophan, and phenylalanine) can result in protein conformational changes that alter affinity to target molecules and potentially increase or decrease binding to inhibitors or stimulators [46]. ROS have also been shown to affect the catalytic rate of kinases and phosphatases, a finding suggesting that ROS have the capacity to ‘fine-tune’ the magnitude and duration of inhibitory and stimulatory signals [46]. Together, these findings suggest a critical role for ROS in improving muscle function acutely, and in stimulating adaptations to exercise training, and that some critical level of ROS production may be needed to limit oxidative damage.

## ROS and oxidative stress

Under conditions of chronic abundance of oxidants relative to antioxidants, oxidative damage can occur. Primary targets of excessive ROS are proteins, lipids, and DNA. As mentioned earlier in this review, oxidizing agents acquire electrons from target molecules. In proteins, this can result in conformational changes, some of which are irreversible. For example, the formation of carbonyl groups on several amino acid residues is an irreversible modification, and affected proteins are targeted for degradation [47,48]. Oxidative stress, then, is a stimulus for protein catabolism.

Lipid molecules can also be targeted by ROS, resulting in them being oxidized, a process called peroxidation. The interaction of a lipid molecule with a radical sets off a self-propagating chain reaction [49], as one radicalized lipid molecule interacts with others, a process that continues until two radicals interact with each other. This process is damaging to cells and tissue, and has been implicated in the development of atherosclerosis, asthma, Parkinson’s disease, and other conditions [49].

Oxidative damage to DNA can take many forms, dependent on the portion of the DNA structure affected, and the specific ROS involved [50-52]. The details of how oxidation affects DNA is beyond the scope of this review, but, briefly, oxidative damage to DNA has been linked to cancer, with its primary role likely being the initiation of carcinogenesis rather than cancer promotion/progression [52].

## Sedentariness, exercise, and oxidative stress

The association between sedentary behavior and chronic disease is well-established, and it may be that oxidative stress is a key process whereby inactivity leads to a number of chronic diseases. Specifically, oxidative stress is known to be associated with aging, cardiovascular disease, and sarcopenia, and it may be that exercise exerts its beneficial effects on each of these disease processes through its effects on redox status.

As discussed previously, the mitochondria are one source of ROS, as superoxide is produced when electrons leak from the ETC. and bind with molecular oxygen. Mitochondrial superoxide production does seem to be increased during exercise relative to a basal state [7], and while factors such as temperature [53] may also influence mitochondrial function and ROS production, it has been suggested that long-term sedentariness produces an environment conducive to high basal levels of superoxide formation. It was noted earlier that mitochondrial ROS production increases less than threefold between rest and exhaustive exercise, while metabolic rate increases approximately 100-fold. Several studies have reported that state IV mitochondrial respiration (resting) is more productive of ROS than state III mitochondrial respiration (exercise) [53-57]. Fisher-Wellman and Neufer [58] explain that the rate at which electrons flow down the ETC. is influenced by energy demand, and not energy availability. As such, sedentariness and the relative lack of adenosine diphosphate (ADP) availability results in a slower transmission of electrons through the ETC., thereby allowing for increased electron leakage. In addition, the concentration of reducing equivalents (e.g., NADH and FADH_2_) influences the cumulative redox state, and an increase in the concentration of reducing equivalents as may be caused by over-nutrition induces a more reduced environment, which subsequently increases the driving force for electron entry into the ETC. at complex I (NADH/NAD+) and complex II (FADH_2_/FAD+) [58]. As a result, though oxygen flux through skeletal muscle is many times higher during exercise, the rate of electron flow through the ETC. is faster due to the increased energy demand (state III respiration). This condition ‘de-stresses’ the mitochondria and permits less time for electron leakage, and as a result mitochondrial ROS production increases relatively little.

People who are chronically inactive also exhibit lower levels of endogenous antioxidants [59-61]. In fact, it appears that the regular inducement of exercise-mediated ROS formation stimulates an adaptation in skeletal muscle that enhances its capacity to manage oxidative stress, a finding consistent with hormesis, the concept that many biological systems respond in a bell-shaped or inverted U-shaped pattern to potential stressors [59]. For example, moderate exercise training alters oxidative homeostasis by reducing basal levels of oxidative damage [60], and by increasing resistance to oxidative stress, mediated largely through increased endogenous antioxidant defenses [60,62]. Jackson et al. [63] found that skeletal muscle contraction-induced ROS activates several identified redox-sensitive transcription factors, including nuclear factor-κB (NF-κB). This transcription factor regulates expression of cell-protective genes during adaptation to exercise [64].

Aerobic exercise training sufficient to improve cardiorespiratory fitness might also be expected to improve oxidative balance via its effects on purine metabolism. As noted previously, hypoxanthine is the substrate for XO, an enzyme that produces xanthine and superoxide. Intensive, and especially anaerobic, exercise has been shown to lead to increased XO activity [25]. Because aerobic exercise training typically increases aerobic capacity and work rate at lactate threshold, it is capable of reducing hypoxanthine levels in the plasma [65], thereby providing less substrate for XO and resulting in less ROS production at any submaximal absolute work rate.

Sirtuin 1 (SIRT1) is an enzyme that functions to remove acetyl groups from proteins involved in cellular regulation and can therefore influence expression of specific genes. Two known targets of SIRT1 are PGC-1a and FOXOs, which are Forkhead box transcription factors, some of which affect antioxidant enzyme expression (e.g., catalase and MnSOD) [66-68]. Exercise training has been shown to increase SIRT1 activity [69], and this may mediate much of the exercise-related improvement in oxidative state. Increased mitochondrial biogenesis and increased mitochondrial density alone may reduce ROS production while at rest by providing new mitochondria to supplement those that are aged or dysfunctional due to oxidative stress, as well as increasing mitochondrial membrane surface area to facilitate diffusion and dissipation of proton gradients, thereby reducing the slowing of electron flow in the ETC. during rest [68]. Additionally, long-term exercise training appears to increase the level of endogenous antioxidants in proportion to the level of ROS production, resulting in a supercompensation that affords protection from their potentially damaging effects [60,62,63].

Good evidence exists showing an inverse relationship between measures of aerobic fitness and oxidative stress [70-72], and, as has been noted, it is likely that much of the protection against oxidative stress afforded by exercise is mediated through improvements in antioxidant capacity. Mitochondrial ROS production is proportional to oxygen flux [6,7], and exercise increases ROS formation in the ETC. Likewise, ROS formation by NOX is also elevated during exercise [18], and it may be that an increase in aerobic capacity may also increase the capacity for ROS formation via these pathways. Conversely, given that improved aerobic fitness would be expected to reduce AMP levels at any given absolute exercise intensity, it may be expected that ROS synthesis by XO would be reduced with an increase in aerobic fitness. It appears that ROS production is increased during exercise, and, as such, it may be that an increase in aerobic capacity is accompanied by an increased capacity for ROS production. However, it also appears that regularly inducing an oxidative environment with exercise results in significant improvements in antioxidant levels and activity. Falone et al. [73] found that recreational runners exhibited greater serum antioxidant capacity and reduced protein carbonyl content before and after exhaustive exercise than untrained subjects. Interestingly, they also reported that, compared to pre-exercise baseline levels, exhaustive exercise resulted in reduced levels of thiobarbituric acid-reactive substances (TBARS) in trained subjects, while resulting in increased levels of TBARS in untrained subjects. (TBARS are formed as a byproduct of lipid peroxidation.) These findings support the conclusion that greater levels of aerobic fitness are accompanied by improved antioxidant capacity such that, though ROS formation is elevated with exercise, indicators of oxidative stress are reduced.

Although it appears that skeletal muscle exhibits the largest exercise-induced enhancement in controlling oxidative stress, other tissues also significantly enhance antioxidant defense mechanisms. For example, Azizbeigi et al. [74] reported that 8 weeks of progressive resistance training improved the antioxidant capacity of erythrocytes mainly through increases in SOD activity. Similarly, Metin et al. [75] reported that erythrocytes in young male soccer players exhibited greater SOD activity than controls. While there may be numerous consequences of these adaptations in the blood (e.g., less lipid oxidation in plasma), one effect that has been identified is that erythrocytes demonstrate better resistance to ROS-induced hemolysis after exercise training [76].

Another example is the liver. This organ has a high metabolic rate at rest, but it is markedly reduced during exercise as blood flow is redirected to the working muscles [77]. As such, a relative ischemia is induced in the liver during exercise. This stimulus results in xanthine dehydrogenase being converted to XO and increased ROS production via the enzyme-catalyzed mechanism described earlier. It is noteworthy that the liver contains much higher levels of XO than does skeletal muscle [58], and this pathway therefore provides a significant proportion of hepatic ROS. Radak et al. [78] showed that in rats, exercise training at 75% of maximum oxygen uptake (*V*O_2max_) for 8 weeks resulted in a significant reduction in ROS production in the liver compared with non-exercised animals. They also reported that reduced GSH levels were higher, and oxidized GSH (GSSG) levels were lower in the trained group than in the sedentary group, reflecting a more favorable redox state.

Redox balance in the brain also appears to respond favorably to exercise training. Regular physical exercise results in an increase in the content of several antioxidants in the brain, including Cu/ZnSOD and glutathione peroxidase, as well as increases in PGC-1a [79]. In addition, exercise appears to stimulate an increase in the production of brain-derived neurotrophic factor (BDNF) [80], though the mechanisms are not fully understood. BDNF is a uniquely versatile substance, and has been shown to influence brain development, neuroplasticity, neurogenesis, and neuron cell survival [81], and BDNF content in the brain has been shown to increase in response to exercise and oxidative stress [82]. It appears that oxidative stress stimulates an increase in BDNF content, which subsequently stimulates increased antioxidant enzyme expression, at least in part via increased NF-κB activation [83,84]. Oxidative stress has been implicated in several disorders of the brain, including Alzheimer’s disease and Parkinson’s disease [85]. A number of studies have found that regular activity provides protection against acquiring these conditions, and the anti-oxidative influence of physical exercise on the brain may provide one important explanation for those findings [85,86]. Importantly, increases in BDNF production appear to depend largely on increased neural activity, which can be induced with exercise, intellectual activity, or reduced caloric intake [87].

## Exercise-induced oxidative environment, ROS signaling, and protein synthesis

It was noted earlier that exercise acutely increases ROS production via increased activity of several enzyme-catalyzed processes. It was also previously noted that several studies have shown that blocking ROS formation associated with exercise also attenuates many of the key adaptations to exercise training. Taken together, these findings suggest that ROS formation is not simply a toxic byproduct of muscle contraction or oxygen metabolism, but is a key participant in stimulating beneficial adaptations to training, and, as such, regularly inducing an oxidative redox state may be important in promoting health. Several recent studies have yielded results that seem to support this position. Nyberg et al. [61] measured arteriovenous GSH and GSSG differences in the legs of young (23 ± 1 years) sedentary, old (66 ± 2 years) sedentary, and old (62 ± 2 years) active men during knee extension exercise. As noted previously, GSH is an antioxidant, and is converted to GSSG in its reduced form, so an increase in GSSG and/or an increase in the GSSG:GSH ratio would indicate a more oxidative redox state. Young sedentary men demonstrated an exercise-associated increase in GSSG and the GSSG:GSH ratio in venous blood that was correlated with intensity. Interestingly, the exercise-related increases did not occur in older sedentary or older active men, though the older active men did display higher levels of GSH and antioxidant enzymes. It appears that the oxidative stress associated with aging chronically activates NF-κB and results in increased antioxidant enzyme activity, but the capacity of aged muscle to stimulate a further increase of ROS during contraction sufficient to measurably alter the GSSG:GSH ratio is significantly reduced compared with young muscle [88-90]. Given the important signaling roles played by ROS (e.g., stimulation of mitochondrial biosynthesis, described previously), it is possible that a reduced capacity to stimulate an oxidative redox state via muscle contraction may contribute to aging and the development of chronic disease.

Recently, it has also been speculated that the oxidative environment that is associated with exercise may be critical for the synthesis of certain proteins, specifically those that exhibit disulphide bonds, many of which are membrane or secretory proteins [91]. Oxidation of cysteine residues leads to the formation of sulfenic acid intermediates, and in close proximity to other oxidized cysteine residues can result in disulphide bonds [92,93]. Said another way, these bonds cannot be formed in a reduced redox state. In fact, Ron and Harding [94] found that the endoplasmic reticulum of sedentary, insulin-resistant rats had significantly fewer disulphide bonds and, therefore, more unfolded proteins than their active, insulin-sensitive counterparts. Therefore, it may be that the transient and significant increase in ROS production and the consequent oxidative shift that results during exercise is necessary to optimize the production of these proteins, and people who are chronically inactive may not provide that environment frequently enough for optimal protein synthesis. If this is true, it provides yet one more additional mechanism whereby regular physical exercise sufficient to produce an oxidative redox state optimizes health.

## Summary

**Figure 1:**
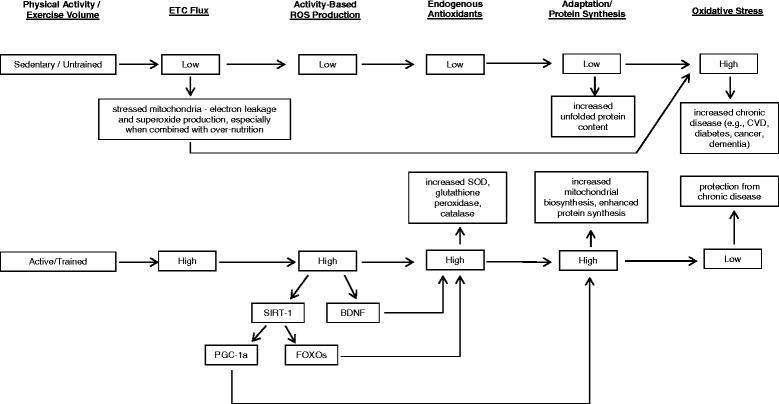
***BDNF*** **brain-derived neurotrophic factor, ETC. electron transport chain,** ***CVD*** **cardiovascular disease,** ***FOXOs*** **forkhead box transcription factors,** ***PGC-1a*** **peroxisome proliferator-activated receptor-gamma coactivator-1 alpha,** ***ROS*** **reactive oxygen species,** ***SIRT-1*** **sirtuin-1,** ***SOD*** **superoxide dismutase.**

Oxidative stress is a consequence of the presence of too many oxidants relative to antioxidants, and can result in cellular or tissue damage, illness, and disease. However, ROS are not entirely toxic, and research evidence indicates important healthy roles for oxidants, including cell signaling, adaptation, and inducing an environment conducive to synthesis of proteins containing disulphide bonds. Several studies report that many of the beneficial adaptations to exercise training are reduced or eliminated when ROS levels are artificially reduced, further supporting the conclusions that regularly inducing an oxidative redox state through exercise is healthful. Habitual sedentariness, especially in combination with over-nutrition, may lead to chronically stressed mitochondria and increased mitochondria-sourced superoxide production, and to reduced levels of endogenous antioxidant enzymes, as well as reduced mitochondrial biosynthesis, all of which foster a vicious cycle that results in an environment that is increasingly oxidative. Conversely, regular exercise appears to produce a hormesis response, whereby the stressor (exercise) induces a transient, systemically oxidative environment that stimulates adaptations enhancing the capacity of tissues throughout the body to neutralize ROS and to more effectively regulate redox balance (see Figure 1). These adaptations provide resistance to development of a number of chronic diseases, and may explain much of the exercise-related improvement in health and longevity cited in many epidemiological studies.

## References

[1] Sies H, Jones DP (2007). Oxidative stress.

[2] Gutowski M, Kowalczyk S (2013). A study of free radical chemistry: their role and pathophysiological significance. Acta Biochim Pol.

[3] Pryor WA (1966). Free radicals.

[4] Xu X, Arriaga EA (2009). Qualitative determination of superoxide release at both sides of the mitochondrial inner membrane by capillary electrophoretic analysis of the oxidation products of triphenylphosphonium hydroethidine. Free Radic Biol Med.

[5] Muller FL, Liu Y, Van Remmen H (2004). Complex III releases superoxide to both sides of the inner mitochondrial membrane. J Biol Chem.

[6] St-Pierre J, Buckingham JA, Roebuck SJ, Brand MD (2002). Topology of superoxide production from different sites in the mitochondrial electron transport chain. J Biol Chem.

[7] Saborido A, Naudi A, Portero-Otin M, Pamplona R, Megias A (2011). Stanozolol treatment decreases the mitochondrial ROS generation and oxidative stress induced by acute exercise in rat skeletal muscle. J Appl Physiol.

[8] Gaitanos GC, Williams C, Boobis LH, Brooks S (1993). Human muscle metabolism during intermittent maximal exercise. J Appl Physiol.

[9] Corbi G, Conti V, Russomanno G, Longobardi G, Furgi G, Filippelli A, Ferrara N (2013). Adrenergic signaling and oxidative stress—a role for sirtuins?. Front Physiol.

[10] Kang SW, Chae HZ, Seo MS, Kim K, Baines IC, Rhee SG (1998). Mammalian peroxiredoxin isoforms can reduce hydrogen peroxide generated in response to growth factors and tumor necrosis factor-a. J Biol Chem.

[11] Tao L, Gao E, Bryan NS, Qu Y, Liu HR, Hu A (2004). Cardioprotective effects of thioredoxin in myocardial ischemia and reperfusion: role of S-nitrosation. Proc Natl Acad Sci U S A.

[12] Yamawaki H, Haendeler J, Berk BC (2003). Thioredoxin: a key regulator of cardiovascular homeostasis. Circ Res.

[13] Lu SC (1999). Regulation of hepatic glutathione synthesis: current concepts and controversies. FASEB J.

[14] Fu XJ, Peng YB, Hu YP, Shi YZ, Yao M, Zhang X (2014). NADPH oxidase 1 and its derived reactive oxygen species mediated tissue injury and repair. Oxidative Med Cell Longev.

[15] Halliwell B, Gutteridge J (2007). Free radicals in biology and medicine.

[16] Cherednichenko G, Zima AV, Feng W, Schaefer S, Blatter LA, Pessah IN (2004). NADH oxidase activity of rat cardiac sarcoplasmic reticulum regulates calcium induced calcium release. Circ Res.

[17] Xia R, Webb JA, Gnall LL, Cutler K, Abramson JJ (2003). Skeletal muscle sarcoplasmic reticulum contains a NADH-dependent oxidase that generates superoxide. Am J Physiol Cell Physiol.

[18] Nathan C, Cunningham-Bussel A (2013). Beyond oxidative stress—an immunologist’s guide to reactive oxygen species. Nat Rev Immunol.

[19] Espinosa A, Leiva A, Pena M, Muller M, Debandi A, Hidalgo C (2006). Myotube depolarization generates reactive oxygen species through NAD(P)H oxidase: ROS-elicited Ca^2+^ stimulates ERK, CREB, early genes. J Cell Physiol.

[20] Hidalgo C, Sanchez G, Barrientos G, Aracena-Parks P (2006). A transverse tubule NADPH oxidase activity stimulates calcium release from isolated triads via ryanodine receptor type 1 S-glutathionylation. J Biol Chem.

[21] Zhao X, Bey EA, Wientjes FB, Cathcart MK (2002). Cytosolic phospholipase A2 (cPLA2) regulation of human monocyte NADPH oxidase activity: cPLA2 affects translocation but not phosphorylation of p67(phox) and p47(phox). J Biol Chem.

[22] Gong MC, Arbogast S, Guo Z, Mathenia J, Su W, Reid MB (2006). Calcium independent phospholipase A2 modulates cytosolic oxidant activity and contractile function in murine skeletal muscle cells. J Appl Physiol.

[23] McCord JM (1985). Oxygen-derived free radicals in postischemic tissue injury. N Engl J Med.

[24] Kalyanaraman B (2013). Teaching the basics of redox biology to medical and graduate students: oxidants, antioxidants and disease mechanisms. Redox Biol.

[25] Radak Z, Asano K, Inoue M, Kizaki T, Oh-Ishi S, Suzuki K (1995). Superoxide dismutase derivative reduces oxidative damage in skeletal muscle of rats during exhaustive exercise. J Appl Physiol.

[26] Sindler AL, Delp MD, Reyes R, Wu G, Muller-Delp JM (2009). Effects of ageing and exercise training on eNOS uncoupling in skeletal muscle resistance arterioles. J Physiol.

[27] Vasquez-Vivar J, Kalyanaraman B, Martasek P, Hogg N, Masters BS, Karoui H (1998). Superoxide generation by endothelial nitric oxide synthase: the influence of cofactors. Proc Natl Acad Sci U S A.

[28] Cosentino F, Luscher TF (1999). Tetrahydrobiopterin and endothelial nitric oxide synthase activity. Cardiovasc Res.

[29] Bevers LM, Braam B, Post JA, van Zonneveld AJ, Rabelink TJ, Koomans HA (2006). Tetrahydrobiopterin, but not l-arginine, decreases NO synthase uncoupling in cells expressing high levels of endothelial NO synthase. Hypertension.

[30] Pacher P, Beckman JS, Liaudet L (2007). Nitric oxide and peroxynitrite in health and disease. Physiol Rev.

[31] Dikalov S (2011). Cross talk between mitochondria and NADPH oxidases. Free Radic Biol Med.

[32] Powers SK, Jackson MJ (2008). Exercise-induced oxidative stress: cellular mechanisms and impact on muscle force production. Physiol Rev.

[33] Bartosz G (2009). Reactive oxygen species: destroyers or messengers?. Biochem Pharmacol.

[34] Toledano MB, Planson A-G, Delaunay-Moisan A (2010). Reining in H(2)O(2) for safe signaling. Cell.

[35] Rhee SG, Kang SW, Jeong W, Chang TS, Yang KS, Woo HA (2005). Intracellular messenger function of hydrogen peroxide and its regulation by peroxiredoxins. Curr Opin Cell Biol.

[36] Bae YS, Oh H, Rhee SG, Yoo YD (2011). Regulation of reactive oxygen species generation in cell signaling. Mol Cells.

[37] Davies MJ (2005). The oxidative environment and protein damage. Biochim Biophys Acta.

[38] Reid MB (2001). Nitric oxide, reactive oxygen species, and skeletal muscle contraction. Med Sci Sports Exerc.

[39] Reid MB (2001). Redox modulation of skeletal muscle contraction: what we know and what we don’t. J Appl Physiol.

[40] Reid MB, Khawli FA, Moody MR (1993). Reactive oxygen in skeletal muscle. III. Contractility of unfatigued muscle. J Appl Physiol.

[41] Gomez-Cabrera MC, Domenech E, Romagnoli M, Arduini A, Borras C, Pallardo FV (2008). Oral administration of vitamin C decreases muscle mitochondrial biogenesis and hampers training-induced adaptations in endurance performance. Am J Clin Nutr.

[42] Ristow M, Zarse K, Oberbach A, Kloting N, Birringer M, Kiehntopf M (2009). Antioxidants prevent health-promoting effects of physical exercise in humans. Proc Natl Acad Sci U S A.

[43] Yfanti C, Akerstrom T, Nielsen S, Nielsen A, Mounier R, Mortensen OH (2010). Antioxidant supplementation does not alter endurance training adaptation. Med Sci Sports Exerc.

[44] Janssen-Heininger YM, Mossman BT, Heintz NH, Forman HJ, Kalyanaraman B, Finkel T (2008). Redox-based regulation of signal transduction: principles, pitfalls, and promises. Free Radic Biol Med.

[45] Hoshi T, Heinemann S (2001). Regulation of cell function by methionine oxidation and reduction. J Physiol.

[46] Corcoran A, Cotter TG (2013). Redox regulation of protein kinases. FEBS J.

[47] Grune T, Reinheckel T, Davies KJ (1997). Degradation of oxidized proteins in mammalian cells. FASEB J.

[48] Levine RL (2002). Carbonyl modified proteins in cellular regulation, aging, and disease. Free Radic Biol Med.

[49] Mylonas C, Kouretas D (1999). Lipid peroxidation and tissue damage. In Vivo.

[50] Szabo C, Ohshima H (1997). DNA damage induced by peroxynitrite: subsequent biological effects. Nitric Oxide.

[51] Burney S, Caulfield JL, Niles JC, Wishnok JS, Tannenbaum SR (1998). The chemistry of DNA damage from nitric oxide and peroxynitrite. Mutat Res.

[52] Cooke MS, Evans MD, Dizdaroglu M, Lunec J (2003). Oxidative DNA damage: mechanisms, mutation, and disease. FASEB J.

[53] Di Meo S, Venditti P (2001). Mitochondria in exercise-induced oxidative stress. Biol Signals Recept.

[54] Kavazis AN, Talbert EE, Smuder AJ, Hudson MB, Nelson WB, Powers SK (2009). Mechanical ventilation induces diaphragmatic mitochondrial dysfunction and increased oxidant production. Free Radic Biol Med.

[55] Herrero A, Barja G (1997). ADP-regulation of mitochondrial free radical production is different with complex I- or complex II-linked substrates: implications for the exercise paradox and brain hypermetabolism. J Bioenerg Biomembr.

[56] Adhihetty PJ, Ljubicic V, Menzies KJ, Hood DA (2005). Differential susceptibility of subsarcolemmal and intermyofibrillar mitochondria to apoptotic stimuli. Am J Physiol Cell Physiol.

[57] Kozlov AV, Szalay L, Umar F, Kropik K, Staniek K, Niedermuller H (2005). Skeletal muscles, heart, and lung are the main sources of oxygen radicals in old rats. Biochem Biophys Acta.

[58] Fisher-Wellman KH, Neufer PD (2012). Linking mitochondrial bioenergetics to insulin resistance via redox biology. Trends Endocrinol Metab.

[59] Radak Z, Chung HY, Goto S (2008). Systemic adaptation to oxidative challenge induced by regular exercise. Free Radic Biol Med.

[60] Urso ML, Clarkson PM (2003). Oxidative stress, exercise, and antioxidant supplementation. Toxicology.

[61] Nyberg M, Mortensen SP, Cabo H, Gomez-Cabrera MC, Vina J, Hellsten Y (2014). Roles of sedentary aging and lifelong physical activity in exchange of glutathione across exercising human skeletal muscle. Free Radic Biol Med.

[62] Sen CK (1995). Oxidants and antioxidants in exercise. J Appl Physiol.

[63] Jackson MJ, Papa S, Bolanos J, Bruckdorfer R, Carlsen H, Elliott RM (2002). Antioxidants, reactive oxygen and nitrogen species, gene induction and mitochondrial function. Mol Aspects Med.

[64] Vasilaki A, Mansouri A, Remmen H, van der Meulen JH, Larkin L, Richardson AG (2006). Free radical generation by skeletal muscle of adult and old mice: effect of contractile activity. Aging Cell.

[65] Teixeira-Lemos E, Nunes S, Teixeira F, Reis F. Regular physical exercise training assists in preventing type 2 diabetes development: focus on its antioxidant and anti-inflammatory properties. Cardiovasc Diabetol. 2011;10:12. doi:10.1186/1475-2840-10-12. http://www.cardiab.com/content/10/1/12. 10.1186/1475-2840-10-12 PMC3041659 21276212

[66] Finkel T, Deng CX, Mostoslavsky R (2009). Recent progress in the biology and physiology of sirtuins. Nature.

[67] Haigis MC, Sinclair DA (2010). Mammalian sirtuins: biological insights and disease relevance. Annu Rev Pathol.

[68] Guarente L, Franklin H (2011). Epstein lecture: sirtuins, aging, and medicine. N Engl J Med.

[69] Navarro-Arevalo A, Canavate C, Sanchez-del-Pino MJ (1999). Myocardial and skeletal muscle aging and changes in oxidative stress in relationship to rigorous exercise training. Mech Ageing Dev.

[70] Campbell PT, Gross MD, Potter JD, Schmitz KH, Duggan C, McTiernan A (2010). Effect of exercise on oxidative stress: a 12-month randomized, controlled trial. Med Sci Sports Exerc.

[71] Nojima H, Watanabe H, Yamane K, Kitahara Y, Sekikawa K, Yamamoto H (2008). Effect of aerobic exercise training on oxidative stress in patients with type 2 diabetes mellitus. Metabolism.

[72] Elosua R, Molina L, Fito M, Arquer A, Sanchez-Quesada JL, Covas MI (2003). Response of oxidative stress biomarkers to a 16-week aerobic physical activity program, and to acute physical activity, in healthy young men and women. Atherosclerosis.

[73] Falone S, Mirabilio A, Pennelli A, Cacchio M, Di Baldassarre A, Gallina S (2010). Differential impact of acute bout of exercise on redox- and oxidative damage-related profiles between untrained subjects and amateur runners. Physiol Res.

[74] Azizbeigi K, Azarbayiani MA, Peeri M, Agha-alinejad H, Stannard S (2013). The effect of progressive resistance training on oxidative stress and antioxidant enzyme activity in erythrocytes in untrained men. Int J Sport Nutr Exerc Metab.

[75] Metin G, Atukeren P, Alturfan AA, Gulyasar T, Kaya M, Gumustas MK (2003). Lipid peroxidation, erythrocyte superoxide-dismutase activity and trace metals in young male footballers. Yonsei Med J.

[76] Blache D, Lussier-Cacan S, Gagnon J, Leon AS, Rao DC, Skinner JS (2007). Effect of exercise training on in vitro LDL oxidation and free radical-induced hemolysis: the HERITAGE Family Study. Antioxid Redox Signal.

[77] Masud MM, Fujimoto T, Miyake M, Watanuki S, Itoh M, Tashiro M (2009). Redistribution of whole-body energy metabolism by exercise: a positron emission tomography study. Ann Nucl Med.

[78] Radak Z, Chung HY, Naito H, Takahashi R, Jung KJ, Kim HJ (2004). Age-associated increase in oxidative stress and nuclear factor kappaB activation are attenuated in rat liver by regular exercise. FASEB J.

[79] Barde YA (1989). Trophic factors and neuronal survival. Neruon.

[80] Stranahan AM, Lee K, Martin B, Maudsley S, Golden E, Cutler RG (2009). Voluntary exercise and caloric restriction enhance hippocampal dendritic spine density and BDNF levels in diabetic mice. Hippocampus.

[81] van Praag H, Kempermann G, Gage FH (1999). Running increases cell proliferation and neurogenesis in the adult mouse dentate gyrus. Nat Neurosci.

[82] Mattson MP, Maudsley S, Martin B (2004). A neural signaling triumvirate that influences ageing and age-related disease: insulin/IGF-1. BDNF and Serotonin Ageing Res Rev.

[83] Mattson MP, Lovell MA, Furukawa K, Markesbery WR (1995). Neurotrophic factors attenuate glutamate-induced accumulation of peroxides, elevation of intracellular Ca^2+^ concentration, and neurotoxicity and increase antioxidant enzyme activities in hippocampal neurons. J Neurochem.

[84] Radak Z, Marton O, Nagy E, Koltai E, Goto S (2013). The complex role of physical exercise and reactive oxygen species on the brain. J Sport Health Sci.

[85] Marques-Aleixo I, Oliveira PJ, Moreira PI, Magalhaes J, Ascensao A (2012). Physical exercise as a possible strategy for brain protection: evidence from mitochondrial-mediated mechanisms. Prog Neurobiol.

[86] Texel SJ, Mattson MP (2011). Impaired adaptive cellular responses to oxidative stress and the pathogenesis of Alzheimer’s disease. Antioxid Redox Signal.

[87] Rothman SM, Mattson MP (2013). Activity-dependent, stress-responsive BDNF signaling and the quest for optimal brain health and resilience throughout the lifespan. Neuroscience.

[88] Vasilaki A, McArdle F, Iwanejko LM, McArdle A (2006). Adaptive responses of mouse skeletal muscle to contractile activity: the effect of age. Mech Ageing Dev.

[89] Cai D, Frantz JD, Tawa NE, Melendez PA, Oh BC, Lidov HG (2004). IKKbeta/NF-kappaB activation causes severe muscle wasting in mice. Cell.

[90] Ji LL (2002). Exercise-induced modulation of antioxidant defense. Ann N Y Acad Sci.

[91] Watson JD (2014). Type 2 diabetes as a redox disease. Lancet.

[92] Drose S, Brandt U (2012). Molecular mechanisms of superoxide production by the mitochondrial respiratory chain. Adv Exp Med Biol.

[93] Brown DI, Griendling KK (2009). Nox proteins in signal transduction. Free Radic Biol Med.

[94] Ron D, Harding HP (2012). Protein-folding homeostasis in the endoplasmic reticulum and nutritional regulation. Cold Spring Harb Perspect Biol.

